# Retrospective study of mechanical complications after cephalomedullary nail implantation from 2019 to 2024 following per-, sub- or intertrochanteric femur fractures

**DOI:** 10.1007/s00402-026-06257-9

**Published:** 2026-03-07

**Authors:** Alexander Blümke, Adaugo Okoro, Aditya Vadgaonkar, Daniel Kühlwein, João Pinheiro, Maximilian Mellinghoff, Frederic Bludau, Andreas Schilder, Svetlana Hetjens, Michael Hackl, Sascha Gravius, Ali Darwich

**Affiliations:** 1 Department of Orthopedics and Trauma Surgery, University Medical Center Mannheim, Medical Faculty Mannheim, Heidelberg University, Mannheim, Germany; 2 Medical Faculty Mannheim, Heidelberg University, Department of Medical Statistics and Biomathematics, Mannheim, Germany

**Keywords:** per-, sub- and intertrochanteric femur fractures, Cephalomedullary nailing, Elderly patients, Osteoporosis, Tip-apex distance, Hemoglobin

## Abstract

**Background:**

Cephalomedullary nail (CMN) fixation is commonly used to treat extracapsular femoral fractures but is associated with mechanical complications, including implant cut-out, cut-in, and material failure. Identifying clinical and radiographic predictors of these complications is critical for improving patient outcomes.

**Methods:**

This retrospective cohort study analyzed 401 patients treated with CMN for per-, sub-, or intertrochanteric fractures between 2019 and 2024. Clinical, laboratory, and radiographic parameters were evaluated using logistic regression analyses to identify predictors of mechanical complications.

**Results:**

Mechanical complications occurred in 7% (n = 28) of patients. The most common complications were cut-out (n = 16, 57%) and cut-in (n = 7, 25%). Significant predictors included increased tip-apex distance (TAD), younger patient age, and the requirement for postoperative blood transfusions. A TAD greater than 37.2 mm substantially elevated the risk of complications.

**Conclusion:**

Mechanical complications after CMN implantation can be significantly reduced by precise implant placement to minimize TAD and careful perioperative management of hemoglobin levels. Implementing these findings into clinical practice can potentially improve surgical outcomes and reduce patient morbidity.

**Supplementary Information:**

The online version contains supplementary material available at 10.1007/s00402-026-06257-9.

## Introduction

Hip fractures are a major public health concern, with over 10 million cases reported globally each year. According to the World Health Organization, hip fractures associated with osteoporosis are projected to increase from 1.7 million in 1990 to 6.3 million by 2050, underscoring the growing burden of fragility fractures [1–4]. Hip fractures are classified as intracapsular or extracapsular, depending on their location relative to the joint capsule. Extracapsular fractures, including pertrochanteric and subtrochanteric types, make up nearly half of all hip fractures in older adults and typically result from low-energy trauma in osteoporotic bone [5–7]. Given the high rates of disability and mortality [8, 9], treatment focuses on stable fixation to enable early mobilization, restore function, and prevent complications [10–12]. In pertrochanteric fractures, preserved femoral head vascularity allows for closed reduction and internal fixation, typically using a cephalomedullary nail (CMN) [7, 13].

Compared to traditional extramedullary devices such as the dynamic hip screw (DHS, DePuy Synthes, Oberdorf, Switzerland), CMNs offer biomechanical advantages, including a shorter lever arm and reduced tensile stress. As a result, CMNs have largely replaced DHS as the standard of care. Modern CMNs, such as the PFNA (DePuy Synthes, Oberdorf, Switzerland) and TFNA (DePuy Synthes, Oberdorf, Switzerland), feature helical blades that enhance rotational and axial stability, especially in osteoporotic bone [14], while dual-screw implants have shown to further reduce complication and reoperation rates in some studies, likely due to improved rotational and axial stability [15, 16].

Despite good overall outcomes, mechanical complications can occur and compromise treatment success. These include implant failure or loss of fixation, such as cut-out or cut-in, blade migration, peri-implant fractures, and nail breakage [17, 18]. Reported rates of mechanical complications range from 2.6 to 13% [19–21]. When failure occurs, revision surgery is often necessary. For example, cut-out typically requires conversion to hemi- or total hip arthroplasty due to femoral head damage [22–24]. These procedures increase morbidity and healthcare costs.

Preventing mechanical complications is essential to reduce patient suffering, length of rehabilitation, and financial burden. Contributing factors include patient characteristics, fracture type, surgical technique, and implant design. Because prior studies are often underpowered and focused primarily on radiographic metrics [21], this study aimed to validate known predictors and expand the analysis to include patient-related risk factors using a large, well-characterized cohort. By quantifying the incidence of mechanical complications after CMN implantation over a five-year period and identifying associated variables, we seek to improve risk stratification, inform surgical decision-making, and ultimately enhance patient outcomes.

## Materials & methods

### Study design and setting

This single-center, retrospective cohort study analyzed 401 patients who underwent CMN implantation for per-, sub-, or intertrochanteric femoral fractures at a university medical center between 2019 and 2024. The study was approved by the local ethics committee (IRB No. 2024–849). Informed consent was waived due to the retrospective analysis of anonymized data.

The primary aim of this study was to evaluate clinical and radiographic outcomes following CMN fixation of proximal femoral fractures. A secondary objective was to identify potential risk factors associated with mechanical complications.

### Study population

Operative records were screened to identify all patients who underwent CMN implantation for proximal femoral fractures between 2019 and 2024. This yielded 414 cases. To ensure a homogeneous study population with low-energy, osteoporotic fractures, patients with high-energy trauma were excluded. All excluded patients were under the age of 55, resulting in the removal of 13 cases. The final study cohort included 401 patients, categorized into two groups: 28 (7%) with mechanical complications and 373 without. Data analysis was performed in 2025, providing a minimum monitoring period of 18 months for all patients to confirm the status of the control group and identify any delayed mechanical complications.

### Definitions

Mechanical complication was defined as either (1) implant cut-out, (2) implant cut-in, (3) lateral protrusion of the blade or screw, (4) bolt loosening or (5) nail breakage.

**Fig. 1:**
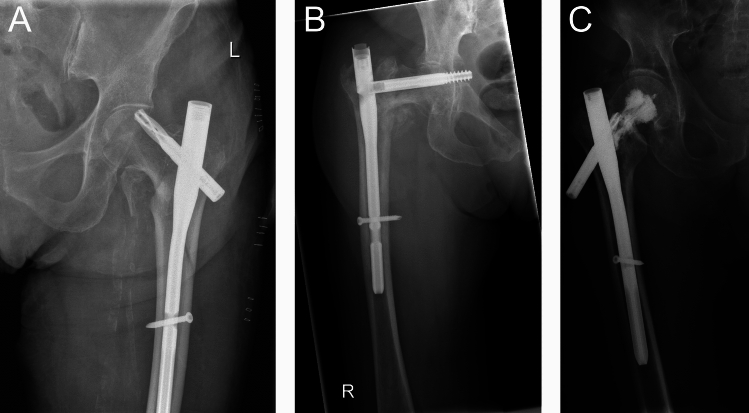
Representative radiographic examples of mechanical complications following intramedullary nailing of proximal femoral fractures. **A** Cut-out: Cranio-lateral penetration of the blade or screw through the femoral head after implantation of a cephalomedullary nail. **B** Cut-in: Central or superomedial migration of the blade or screw into the acetabulum, or in this case, even extending into the lesser pelvis. **C** Lateral protrusion: Lateral migration of the blade or screw beyond the greater trochanter

“Cut-out” was referred to as the cranio-lateral penetration of the blade or screw following implantation of a CMN as has been defined elsewhere [25, 26] (Fig. 1A). This is usually associated with a loss of fracture reduction, resulting in varus collapse of the head-neck fragment.

“Cut-in” (often used synonymously with “cut-through” throughout literature), as defined elsewhere [27, 28], was referred to as the central or superomedial migration of the blade or screw into the acetabulum (Fig. 1B).

“Lateral protrusion” was referred to as the lateral migration of the blade or screw following CMN implantation as defined elsewhere [26] and may cause mechanical irritation of the skin and soft tissues (Fig. 1C). Additionally, fracture and fixation stability may be compromised.

### Data collection

Clinical and laboratory data were extracted from the hospital information system and laboratory database. Over 50 parameters, grouped into preoperative, intraoperative, and postoperative parameters were systematically assessed, as summarized in Table 1.

**Table 1:** Overview and univariate analysis of parameters, categorized as preoperative, intraoperative, and postoperative

Variable	All patients (n = 401)	No mechanical complication (n = 374)	Mechanical complication (n = 27)	p-value	Odds ratio	95% confidence interval
**Preoperative parameters**						
*Demographics*						
Sex (Female: Male)	252: 149	235: 138 (63.0%: 37.0%)	17: 11 (60.7%: 39.3%)	0.698	0.85	0.38–1.89
Age at surgery	81.7	82.0	76.9	**0.036**	0.96	0.93–1.00
*Fracture related*						
Fracture localization (Left: Right)	175: 226	166: 207 (44.5%: 55.5%)	9: 19 (32.1%: 67.9%)	0.262	0.62	0.27–1.42
Fracture type (Classified acc. to the AO/OTA classification system)	399	372	27	0.294		
31A1	184	175 (47%)	9 (33.3%)			
31A2	189	176 (47.3%)	13 (48.2%)			
31A3	15	12 (3.2%)	3 (11.1%)			
31B3	10	8 (2.2%)	2 (7.4%)			
32A1	1	1 (0.3%)	0			
Radiographic lateral wall integrity (> 20.5 mm: ≤ 20.5 mm)	216: 184	205: 167 (55.1%: 44.1%)	11: 17 (39.3%: 60.7%)	0.110	0.53	0.24–1.16
Pre-facture mobility status	253	227	26	0.669		
Bedridden	6	6 (2.6%)	0			
Wheelchair	6	6 (2.6%)	0			
Able to stand	1	0	1 (3.8%)			
Walking frame	1	1 (0.5%)	0			
Walker	106	100 (44.1%)	6 (23.1%)			
Walking stick	2	2 (0.9%)	0			
Without assistive devices	131	112 (49.3%)	19 (73.1%)			
*Comorbidities*						
Number of pre-existing medical conditions	7	7	6	0.186	0.94	0.86–1.03
Heart failure (Yes: No)	52: 345	44: 327 (11.9%: 88.1%)	8: 18 (30.8%: 69.2%)	**0.006**	3.45	1.43–8.33
Cerebrovascular diseases (Yes: No)	85: 312	84: 287 (22.6%: 77.4%)	1: 25 (3.8%: 96.2%)	0.058	0.14	0.02–1.06
Chronic lung diseases (Yes: No)	24: 373	20: 351 (5.4%: 94.6%)	4: 22 (16%: 84%)	**0.042**	3.33	1.04–11.1
Charlson Comorbidity Index (CCI)	6	6	5	0.145	0.86	0.71–1.05
American Society of Anesthesiologists (ASA) risk classification score	3	3	3	0.145	0.64	0.37–1.16
Body mass index (BMI, kg/m^2^)	24.7	24.9	23.9	0.244	0.94	0.84–1.05
Number of prescribed medications at admission	6	6	8	**0.033**	1.10	1.01–1.20
Baseline osteoporosis therapy (Yes: No)	104: 291	94: 274 (25.5%: 74.5%)	10: 17 (37%: 63%)	0.312	1.54	0.67–3.57
Specific osteoporosis therapy (Yes: No)	14: 381	13: 355 (3.5%: 96.5%)	1: 26 (3.7%: 96.3%)	0.934	1.09	0.14–8.33
*Laboratory values*						
Hemoglobin level at admission (g/dL)	11.9	11.9	12.2	0.469	1.08	0.87–1.35
Hematocrit ratio at admission (%)	34.7	34.7	34.9	0.934	1.00	0.93–1.08
Leukocyte count at admission (10^9^/L)	11.0	11.0	11.6	0.432	1.03	0.95–1.12
Albumin level at admission (g/L)	32.8	32.6	35.1	0.120	1.10	0.98–1.24
**Intraoperative parameters**						
*Surgical timing*						
Surgical duration (minutes)	51.4	50.7	62.9	0.104	1.01	1.00–1.02
Time from admission to implantation (hours)	14.2	14.2	14.8	0.867	1.00	0.97–1.03
*Surgical technique*						
Surgical reduction technique (Open: Closed)	32: 366	30: 343 (8%: 92%)	2: 23 (8%: 92%)	0.959	0.96	0.22–4.29
Application of cerclage wires (Yes: No)	26: 375	24: 349 (6.4%: 93.6%)	2: 26 (7.1%: 92.9%)	0.843	1.16	0.26–5.26
Cement augmentation (Yes: No)	43: 358	40: 333 (10.7%: 89.3%)	3: 25 (10.7%: 89.3%)	0.590	1.50	0.34–6.58
*Implant characteristics*						
Nail model (PFNA vs. TFNA)	332: 67	311: 62 (83.4%: 16.6%)	5: 21 (19.2%: 80.8%)	0.663	0.80	0.29–2.21
CMN length (200–240 mm: 340–420 mm)	375: 23	352: 20 (94.6%: 5.4%)	23: 3 (88.5%: 11.5%)	0.205	0.43	0.12–1.56
CMN diameter (mm)	10.4	10.4	10.4	0.532	1.21	0.66–2.22
CMN blade/screw length (mm)	99.7	99.6	100.8	0.489	1.02	0.97–1.07
Distal locking screw length (mm)	36.6	36.7	35.8	0.462	0.95	0.83–1.09
Use of second distal locking screw (Yes: No)	10: 391	10: 363 (2.7%: 97.3%)	0: 28 (0%: 100%)	0.981		
*Anesthesia*						
Type of anesthesia (General anaesthesia: Spinal anesthesia)	219: 128	205: 118 (63.5%: 36.5%)	14: 10 (58.3%: 41.7%)	0.524	0.52	0.07–4.00
**Postoperative parameters**						
Weight-bearing status (Full: Partial)	384:08:00	359: 8 (97.8%: 2.2%)	25:0	0.983		
*Radiographic outcomes*						
Contralateral CCD angle (°)	132	132.0	132.5	0.676	1.01	0.96–1.07
CCD angulation after CMN implantation (°)	129.1	129.3	126.1	0.199	0.98	0.96–1.01
Difference in CCD angle (implant angle vs. contralateral angle, °)	5.7	5.7	5.7	0.867	0.99	0.90–1.09
Reduction quality (Good: Acceptable: Poor) p-value and OR compare Good vs. Poor	267: 103: 20	251: 97: 17 (68.8%: 26.6%: 4.6%)	16: 6: 3 (64%: 24%: 12%)	0.279	0.36	0.096–1.36
Tip-Apex Distance – anteroposterior projection, TAD_ap_ (mm)	11.4	11.3	14.1	**0.003**	1.12	1.04–1.21
Tip-Apex Distance – lateral projection, TAD_lat_ (mm)	12.0	11.9	13.1	0.113	1.07	0.99–1.16
Tip-Apex Distance – anteroposterior + lateral, TAD (mm)	23.4	23.1	27.7	**0.007**	1.06	1.02–1.10
Calcar Tip-Apex Distance – anteroposterior projection, CalTAD_ap_ (mm)	18.5	18.4	19.4	0.429	1.03	0.96–1.12
Calcar Tip-Apex Distance – anteroposterior + lateral, CalTAD (mm)	30.4	30.3	32.5	0.138	1.04	0.99–1.09
Blade position acc. to Cleveland zones	386	362	24	0.889		
1 / 2 / 3	57 / 156 / 57	53 (14.6%) / 148 (40.9%) / 55 (15.2%)	4 (16.7%) / 8 (33.3%) / 2 (8.3%)			
4 / 5 / 6	21 / 81 / 9	18 (4.9%) / 76 (21%) / 8 (2.2%)	3 (12.5%) / 5 (20.8%) / 1 (4.2%)			
7 / 8 / 9	1 / 2 / 2	0 / 2 (0.6%) / 2 (0.6%)	1 (4.2%) / 0 / 0			
Postoperative neck angulation ≤ 5° (Yes: No)	323: 67	306: 59 (83.8%: 16.2%)	17: 8 (68%: 32%)	**0.037**	0.39	0.16–0.94
Fracture type acc. to the Kyle classification (Stable: Unstable)	309: 82	290: 78 (78.8%: 21.2%)	19: 4 (82.6%: 17.4%)	0.603	1.33	0.44–4.00
Stability score acc. to Lee	5.3	5.3	5.4	0.840	1.03	0.80–1.32
*Functional outcomes*						
Barthel Index (3.–7. POD)	41.2	40.9	47.7	0.272	1.01	0.99–1.04
*Complications and interventions*						
Postoperative blood transfusion (Yes: No)	144: 244	128: 236 (35.2%: 64.8%)	16: 8 (66.6%: 33.3%)	**0.006**	3.45	1.43–8.33
Intensive care unit (ICU) admission (Yes: No)	47: 351	43: 330 (11.5%: 88.5%)	4: 21 (16%: 84%)	0.453	1.54	0.50–4.76
*Laboratory values*						
Hemoglobin levels (0.-2. POD, g/dL)	9	9.0	8.3	**0.028**	0.68	0.48–0.96
Hematocrit ratio (0.-2. POD, %)	26.2	26.3	23.6	**0.003**	0.83	0.73–0.94
Leukocyte count (3.-6. POD, 10^9^/L)	8.8	8.7	9.1	0.617	1.03	0.92–1.15
CRP level (3.-6. POD, mg/L)	87.7	87.7	87.0	0.750	1.00	0.99–1.01
Difference between preoperative and postoperative Hb levels (g/dL)	-2.9	-2.9	-3.7	**0.034**	1.35	1.02–1.82

**Fig. 2:**
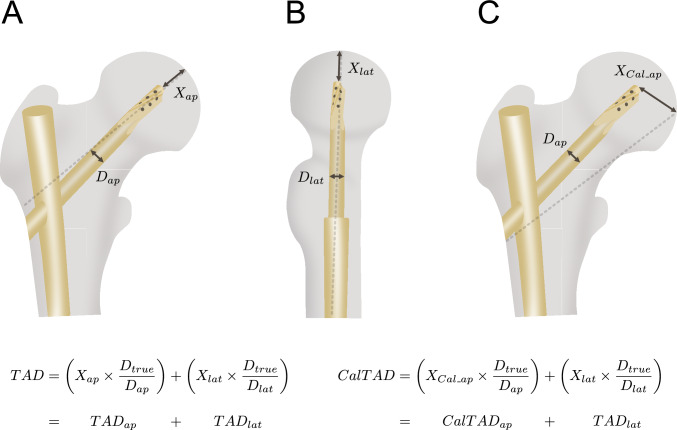
Measured radiographic parameters. Schematic illustration adapted from Chua YP et al. [46]., depicting the calculation of the postoperative tip-apex distance (TAD), defined as the sum of the TAD in the anteroposterior (TAD_ap_) and lateral (TAD_lat_) views. In both the ap view (**A**) and lateral view (**B**), the distance between the tip of the blade or screw and the apex of the femoral head (X_ap_ and X_lat_) is measured. Each measurement is then corrected using the ratio of a known true reference distance (D_true_) to the corresponding radiographic distance (D_ap_ or D_lat_). **C** The calcar-referenced TAD (CalTAD) was calculated similarly: a line was drawn from the lateral end of the blade/screw along the femoral calcar to the femoral head. From there, the distance (X_Cal_ap_) was measured and corrected using the same method as above

Radiographic data were obtained from the hospital’s picture archiving and communication system (PACS). All measurements were performed on digital radiographs. No additional imaging was obtained specifically for this study. Fracture classification followed the AO/OTA system. Additionally, postoperative radiographs were also evaluated to determine specific radiographic measurements, as detailed in Table 1 and included measurement of the tip-apex distance (TAD) and calcar-referenced tip-apex distance (CalTAD), as well as evaluation of implant position using the Cleveland classification. TAD and CalTAD were determined by summing the distances from the tip of the lag screw to the apex of the femoral head on both anteroposterior (TAD_ap_) and lateral (TAD_lat_) radiographs as pointed out in Fig. 2. All measurements were corrected using a known reference value to correct for image magnification. The Cleveland classification was used to describe the spatial position of the lag screw within the femoral head (Fig. 2D). Based on these measurements the stability score according to Lee et al. was determined as described elsewhere [29].

The type of fracture and measurement was determined by two of the authors (AB and AO) through independent review of the PACS images. In case of disagreement, the senior author (AD) assessed the radiographs, and the fracture type or measurement was determined by consensus. To assess inter-rater reliability for radiographic measurements, a blinded secondary review of 60 cases (15% of the total cohort) was performed for TAD and CalTAD. Reliability was evaluated using the Intraclass Correlation Coefficient (ICC) with a two-way random-effects model and absolute agreement for single measures [ICC(2,1)]. Measurement agreement and potential bias were further visualized through Bland–Altman plots.

Patient age at surgery, surgical duration, time from admission to surgery, the difference between preoperative and postoperative hemoglobin values, and total hospitalization duration were calculated. Postoperative hemoglobin concentrations were determined as the lowest value between 4 h postoperatively up to the 2nd postoperative day (POD).

Patients’ osteoporosis management at admission was assessed. Baseline osteoporosis therapy was defined as supplementation with vitamin D and/or calcium. Specific osteoporosis therapy was defined as pharmacological treatment, including bisphosphonates, denosumab, selective estrogen receptor modulators, or osteoanabolically active substances such as romosozumab and teriparatide. Classifications were made according to the current recommendations of the National Osteoporosis Foundation (NOF) and relevant literature.

Patient health at admission was assessed using the American Society of Anesthesiologists (ASA) risk classification and the Charlson Comorbidity Index (CCI).

For patients who sustained a fracture while already hospitalized, the time of admission was defined as the time of fracture diagnosis. The time of discharge was defined as the moment the patient either exited the hospital or was transferred to another department within the hospital.

### Outcomes

The primary outcome of this study was the occurrence of mechanical complications following CMN implantation as defined above. In addition to complication rates, several secondary outcomes were assessed. Radiographic outcomes included TAD, CalTAD, and implant positioning according to the Cleveland classification. Functional recovery at discharge was evaluated using the Barthel Index. Clinical outcomes included postoperative hemoglobin levels, the necessity for transfusion with packed red blood cells (PRBCs), inflammatory markers such as CRP and leukocyte count, as well as admission to the intensive care unit (ICU) during the hospital stay.

### Statistical analysis

#### Univariate analysis

Univariate statistical analyses were conducted using SAS (Version 9.4, SAS Institute Inc., Cary, NC, USA). Descriptive statistics such as mean, standard deviation, median, and interquartile ranges were calculated, depending on the data type and distribution. The normal distribution was analysed using the Shapiro–Wilk test. For categorical variables, frequencies and proportions were reported, and comparisons were made using Chi-square or Fisher’s exact test. Continuous variables were compared using t-tests (in the case of normally distributed data) or Mann–Whitney U tests. Furthermore, the optimal cut-off point was estimated using the Youden index (sensitivity + specificity -1). All data visualizations, including bar charts, box plots, and scatter plots, were generated using GraphPad Prism version 10.0 (GraphPad Software, San Diego, CA, USA).

#### Multivariate analysis

To identify independent predictors of mechanical complications, a multivariate logistic regression model was constructed to evaluate several clinical and radiographic factors simultaneously. This multivariate approach was utilized to determine the specific impact of each variable on the likelihood of mechanical complication while adjusting for potential confounders. Variables demonstrating statistical significance in the initial univariate analysis were entered into a backward stepwise selection process using the PROC LOGISTIC procedure in SAS Version 9.4 (SAS Institute Inc., Cary, NC, USA). The backward selection algorithm began with a comprehensive model containing all potential predictor variables identified from the univariate phase. From this starting point, the procedure systematically evaluated the contribution of each factor and successively removed those that made the least statistically significant contribution to the model's predictive power. This iterative process of removal and re-evaluation continued until no remaining variables could be excluded without significantly impairing the overall quality or discriminative ability of the model.

## Results

**Fig. 3:**
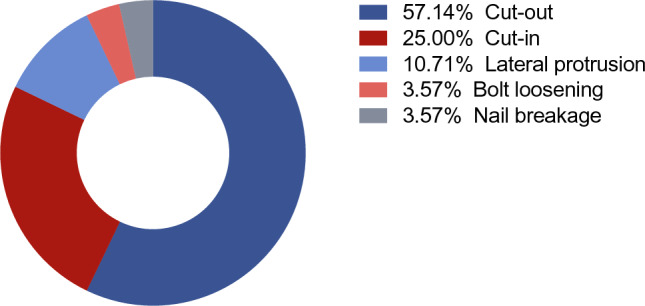
Descriptive analysis. The most frequent complications were cut-out (n = 16) and cut-in (n = 7)

A total of 401 patients were included in the final analysis, of whom 28 (7%) experienced mechanical complications. These complications comprised cut-out (n = 16), cut-in (n = 7), lateral protrusion (n = 3), bolt loosening (n = 1), and nail breakage (n = 1) (Fig. 3). The median age was 84 years (IQR 75—89), and 62.8% of patients were female. The most common recorded comorbidity was hypertension (60.8%). Osteoporosis was noted as a pre-existing condition in 23.4% of cases at the time of admission. The median Charlson Comorbidity Index was 5. Preoperative physical status, as evaluated using the ASA classification, was categorized as ASA I in 4.0% of patients, ASA II in 21.6%, ASA III in 59.4%, and ASA IV in 15.0%.

### Univariate analysis

**Fig. 4:**
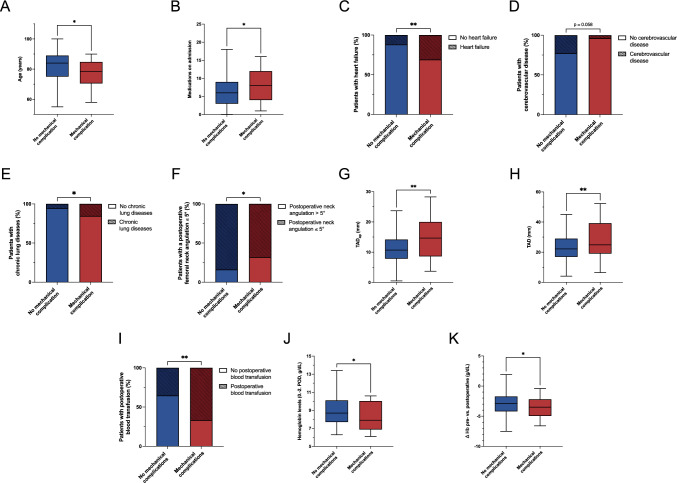
Univariate analysis of clinical and perioperative parameters in the control group versus the complication group. **A** Age at surgery. **B** Number of medications on admission. **C** Proportion of patients with heart failure among pre-existing conditions. **D** Proportion of patients with cerebrovascular disease among pre-existing conditions. **E** Proportion of Patients that received a postoperative blood transfusion. **F** Proportion of Patients with a femoral neck angulation above or below 5° postoperatively. **G** Tip-apex distance in the anteroposterior view (TAD_ap_). **H** Tip-apex distance, combined anteroposterior and lateral views (TAD). **I** Duration of CMN implantation. Statistical analysis was performed using Student’s t-test or chi-square test, as appropriate (*p < 0.05, **p < 0.01)

Univariate analysis revealed that many parameters did not differ significantly between the complication and no-complication groups (Table 1). Nonetheless, several parameters were significantly associated with mechanical complications (Fig. 4). Patients in the control group were significantly older compared to those with complications (82 vs. 77 years, p = 0.036; Fig. 4A). However, the complication group had a significantly higher number of preoperative medications (6 vs. 8; p = 0.033; Fig. 4B). Pre-existing heart failure was significantly more common in the complication group (11.9% vs. 30.8%; p = 0.006; Fig. 4C), as well as chronic lung diseases (5.4% vs. 16%; p = 0.042; Fig. 4D). Cerebrovascular disease tended to be less frequent in the complication group (22.6% vs. 3.8%; p = 0.058; Fig. 4E). Postoperatively, the proportion of patients with a femoral neck angulation > 5° was significantly higher among patients with complications (16.2% vs. 32.0%; p = 0.037, Fig. 4F). Inter-rater reliability was 0.933 (95% CI: 0.904–0.965) for TAD and 0.860 (95% CI: 0.817–0.931) for CalTAD. Corresponding Bland–Altman plots are provided in the supplemental information. Both TAD_ap_ (11 vs. 14 mm; p = 0.003; Fig. 4G) and TAD (23 vs. 28 mm; p = 0.007; Fig. 4H) were significantly greater in the complication group. Furthermore, patients with complications received significantly more postoperative blood transfusions (35.2% vs. 66.6%; p = 0.006; Fig. 4I), correlating with a lower postoperative hemoglobin value (9.0 vs. 8.3 g/dL; p = 0.028; Fig. 4J) and a greater hemoglobin decrease compared to preoperative values (-2.9 vs. -3.7 g/dL; p = 0.034; Fig. 4K).

**Fig. 5:**
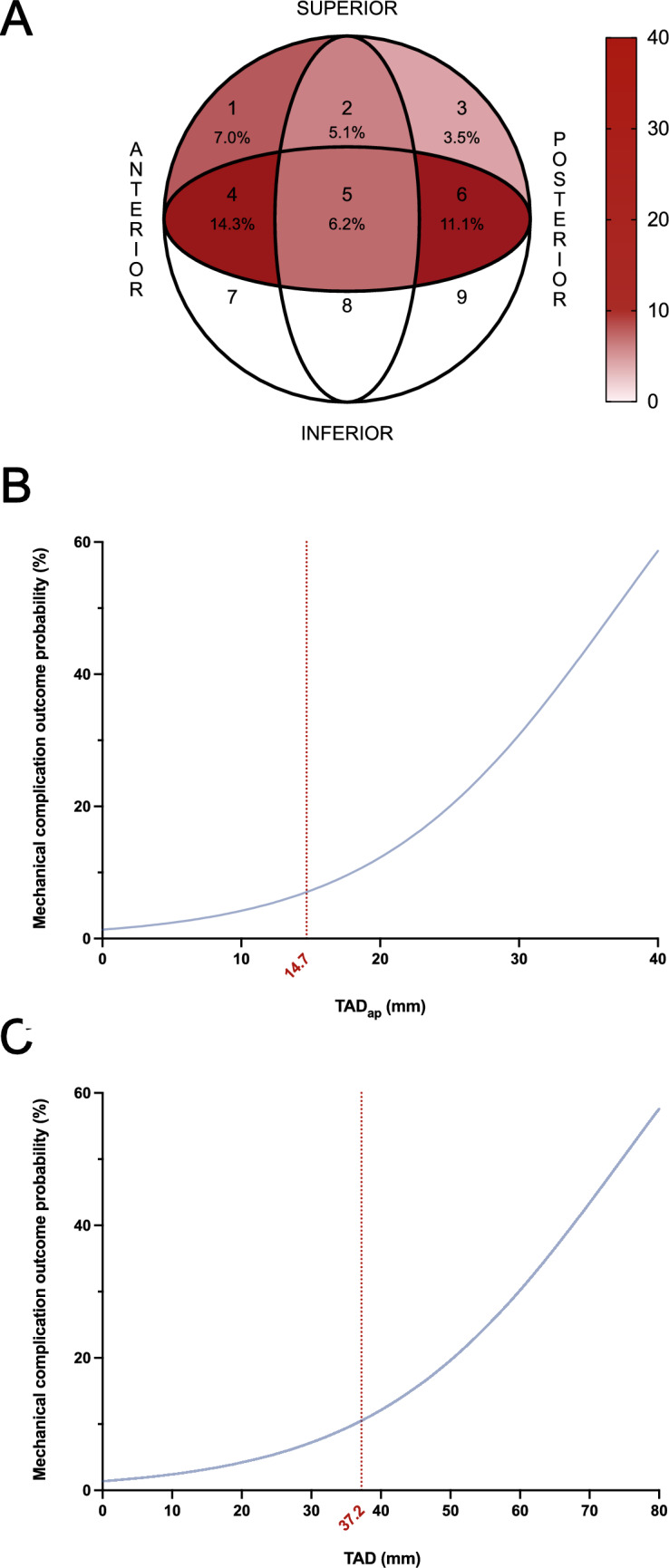
Complication frequency and outcome probability. **A** Blade positions of the cephalomedullary nail within the femoral head, categorized according to the Cleveland zones, along with the relative frequency of mechanical complications in each zone. **B** Logistic regression analysis of TAD_ap_, showing a complication risk threshold at 14.7 mm. **C** Logistic regression analysis of TAD, indicating a complication risk threshold at 37.2 mm

Next, complication frequency based on the location of the blade/screw tip in the femoral head according to the Cleveland zones was visualized in Fig. 5A. The highest complication frequencies were observed in zone 4 and zone 6. Next, the mechanical complication outcome probability was stratified based on the TAD_ap_ (Fig. 5B), and TAD (Fig. 5C), using a logistic regression and calculated a cutoff value. Cutoff for TAD_ap_ was 14.7 mm while for TAD was 37.2 mm.

Finally, we constructed a multivariate logistic regression model (Table 2) including eleven predictors: age at surgery, heart failure, chronic lung disease, number of prescribed medications at admission, TAD_ap_, TAD, postoperative neck angulation, postoperative blood transfusion, hemoglobin levels, hematocrit ratio and difference beween pre- and postoperative Hb levels. The backward logistic regression analysis identified three factors: TAD_ap_, postoperative blood transfusion, and age at surgery. The corresponding β coefficients were 0.113, -0.727, and -0.049, respectively, yielding odds ratios of 1.12, 0.23, and 0.95. The model demonstrated good discriminative ability, with an area under the receiver operating characteristic curve (AUC) of 0.785.

**Table 2:** Stepwise multivariate logistic regression model. Results of the stepwise multivariate logistic regression analysis identifying independent risk factors for mechanical complications

Step	Predictor	β Coefficient (SE)	Odds Ratio (95% CI)	Wald χ^2^	p-value
1	Tip-Apex Distance—anteroposterior projection, TAD_ap_ (mm)	0.116 (0.046)	1.12 (1.03–1.23)	6.51	0.011
2	Postoperative blood transfusion	-0.706 (0.261)	0.24 (0.09–0.68)	7.33	0.007
3	Age at surgery (years)	-0.048 (0.025)	0.95 (0.91–1.00)	3.90	0.048
	Model statistics		AUC = 0.783	χ^2^(3) = 21.54	< 0.001
	Sample size (complete cases)		n = 232		

## Discussion

The primary aim of this study was to identify clinical and radiographic risk factors associated with mechanical complications following CMN implantation for extracapsular femoral fractures. While many previous investigations have focused primarily on radiographic parameters, clinical and patient-related factors are often underrepresented in the analysis of implant failure. In this retrospective cohort of 401 patients treated between 2019 and 2024, mechanical complications occurred in 7% of cases. Cut-out was the most frequent complication, followed by cut-in. Reported incidence range from 3% [30] up to 21% [19].

Several clinical and radiographic factors were identified in the univariate analysis as significantly associated with mechanical complications. Patients in the complication group were significantly younger; however they had a higher number of preoperative medications, suggesting greater frailty and multimorbidity. In particular, heart failure was more frequently observed among patients with complications, potentially reflecting reduced physiological reserves and compromised tissue perfusion, which may affect postoperative recovery and bone healing [31]. Cerebrovascular disease was significantly less prevalent in the complication group (3.8% vs. 22.6%). While this finding may appear counterintuitive, it could hypothetically reflect lower levels of mobility and reduced mechanical loading on the implant in patients with neurological impairments. In these less active individuals, the lack of repetitive weight-bearing might prevent a suboptimal implant from physically failing, potentially masking a complication that would otherwise manifest under higher stress. Conversely, this mechanical logic may explain why younger age was identified as an independent predictor of failure. Relatively younger geriatric patients likely exhibit higher activity levels, subjecting the cephalomedullary construct to greater axial forces and repetitive mechanical stress. Such increased loading might trigger failure that would remain latent in sedentary patients, especially when the tip-apex distance is suboptimal.

Radiological parameters such TAD and TAD_ap_ were significantly higher in the complication group compared to the control group. This finding aligns well with numerous previous studies that have established an increased TAD as one of the strongest predictors for mechanical failure, particularly cut-out and cut-in events [32–34]. A TAD greater than 35 mm has been associated with a markedly increased risk of implant migration and fixation failure, as the implant tip lies further from the subchondral bone, resulting in reduced resistance to rotational and axial forces [33]. In our cohort, logistic regression identified a complication risk threshold of 14.7 mm for the AP projection (TAD_ap_) and 37.2 mm for the combined TAD, emphasizing the importance of meticulous implant placement. While the relationship between increased TAD and implant failure is widely recognized, our study serves to validate these findings within a large, modern cohort of over 400 patients. By identifying specific risk thresholds we provide contemporary, data-driven metrics that reinforce the critical importance of precise implant placement, even when utilizing modern helical blade designs intended to enhance stability [35].

Although previously suggested as relevant predictors, neither the CalTAD nor the stability score according to Lee et al. showed a significant association with mechanical complications in our cohort [29, 33]. While both parameters have been proposed as more nuanced radiographic tools to assess implant positioning and fracture stability, their predictive value could not be confirmed in our analysis. One possible explanation is that CalTAD, although conceptually useful, may be more susceptible to measurement variability due to the anatomical variability of the calcar and projectional differences in radiographs. Similarly, the stability score according to Lee et al., which integrates several radiographic dimensions into a composite measure of stability, may not capture the full complexity of patient-specific risk factors or intraoperative variables that influence implant performance.

Interestingly, patients in the complication group required significantly more often postoperative transfusions of packed red blood cells (PRBC) compared to the control group. This was accompanied by both lower postoperative hemoglobin values and a greater perioperative drop in hemoglobin levels. These findings may point toward a relevant systemic factor contributing to mechanical failure. While causality cannot be established in this retrospective design, significant perioperative blood loss and the resulting anemia may impair tissue oxygenation and bone healing capacity which are important in fracture consolidation and implant integration [36, 37]. As postoperative blood transfusion often serves as a clinical proxy for surgical complexity and intraoperative blood loss, its identification as an independent predictor may suggest that technically demanding cases or those involving poor bone quality carry a higher intrinsic risk of mechanical failure. Moreover, the greater hemoglobin loss observed in the complication group may reflect factors such as poorer bone quality or an increased intraoperative bleeding tendency rather than differences in surgical duration, which did not differ significantly between groups.

Biological factors such as perioperative hemoglobin levels, systemic inflammation, or markers of frailty are still underrepresented in the literature on mechanical complications after CMN fixation. The majority of existing studies focus predominantly on radiographic parameters, while clinical or physiological variables are often neglected or treated as secondary [38].

Nevertheless, there is growing evidence that systemic biological factors may substantially influence patient outcomes following hip fracture surgery. Several studies have demonstrated that preoperative anemia is associated with poorer functional recovery and increased complication rates in elderly patients undergoing proximal femoral fixation [39, 40]. Perioperative blood loss may further exacerbate these effects by impairing tissue oxygenation and delaying bone healing. In intertrochanteric fractures, low hemoglobin levels have been linked to impaired fracture union and, in some cases, mechanical failure of the implant [41, 42]. Additionally, moderate to severe anemia has been associated with a higher incidence of systemic complications, such as delirium, venous thromboembolism, and cardiac events, highlighting the broader physiological burden that may indirectly compromise implant performance [41, 43].

It remains unclear whether increased transfusion requirements, as observed in our complication group, reflect more extensive intraoperative blood loss, technically complex surgical procedures, or underlying patient-specific vulnerabilities such as osteopenia or frailty. Moreover, transfusions themselves are not without risk; they may provoke immunomodulatory effects [44], increase susceptibility to infection [45], and introduce hemodynamic instability, all of which can interfere with bone regeneration and implant integration. Our findings underscore the need to incorporate biological parameters into predictive models of mechanical failure and to consider strategies such as preoperative anemia correction and blood-sparing techniques as potential avenues for reducing complication rates.

This study has several limitations that should be acknowledged. First, its retrospective single-center design inherently introduces the possibility of selection bias and limits the generalizability of findings to other clinical settings with different patient populations or surgical protocols. Second, while over 50 parameters were collected and analyzed, some relevant variables, such as bone mineral density (BMD), vitamin D status, or nutritional markers, were not routinely available and thus could not be included in the analysis. These biological markers may have provided additional insight into the systemic factors contributing to mechanical failure. To account for these missing biological markers, we utilized validated clinical indicators, including the ASA classification, the Charlson Comorbidity Index, and the total number of preoperative medications, as proxies for patient frailty and baseline health. Third, although we analyzed fracture patterns, reduction quality, and lateral wall integrity, the inclusion of these technical variables did not significantly improve the discriminative ability of our multivariate model. Fourth, functional outcomes beyond discharge were not assessed, and thus the long-term clinical significance of the mechanical complications remains unclear. Lastly, while transfusion requirement emerged as an independent predictor of complications, causal relationships cannot be inferred, and confounding by unmeasured variables (e.g., intraoperative blood loss, surgical difficulty) cannot be excluded.

## Conclusions

In summary, this study highlights both radiographic and clinical risk factors associated with mechanical complications following cephalomedullary nailing for extracapsular femoral fractures. Tip-apex distance, younger patient age, and perioperative hemoglobin loss emerged as key predictors of mechanical complications. The results highlight the clinical relevance of addressing both operative technique and systemic patient factors as part of a comprehensive strategy to optimize surgical outcomes.

## Supplementary Information

Below is the link to the electronic supplementary material.

Supplementary file1 (PDF 65 KB)

## Data Availability

The datasets used and/or analyzed during the current study are available from the corresponding author on reasonable request.

## Abbreviations

ASA: American Society of Anesthesiologists
AUC: Area under the receiver operating characteristic curve
BMD: Bone mineral density
BMI: Body mass index
CalTAD: Calcar-referenced tip-apex distance
CCI: Charlson Comorbidity Index
CMN: Cephalomedullary nail
CRP: C-reactive protein
DHS: Dynamic hip screw
DXA: Dual-energy X-ray absorptiometry
ICU: Intensive care unit
IQR: Interquartile range
NOF: National Osteoporosis Foundation
PACS: Picture archiving and communication system
PFNA: Proximal Femoral Nail Antirotation
POD: Postoperative day
PRBC: Packed red blood cells
SD: Standard deviation
TAD: Tip-apex distance
TAD_ap_: Tip-apex distance (anteroposterior view)
TAD_lat_: Tip-apex distance (lateral view)
TFNA: Trochanteric femoral nail advanced
WHO: World Health Organization
