# Supplementary figures and images for: Retrospective study of mechanical complications after cephalomedullary nail implantation from 2019 to 2024 following per-, sub- or intertrochanteric femur fractures
Source: Arch Orthop Trauma Surg. 2026 Mar 7;146(1):100. doi: 10.1007/s00402-026-06257-9 (PMC12966185; doi:10.1007/s00402-026-06257-9)

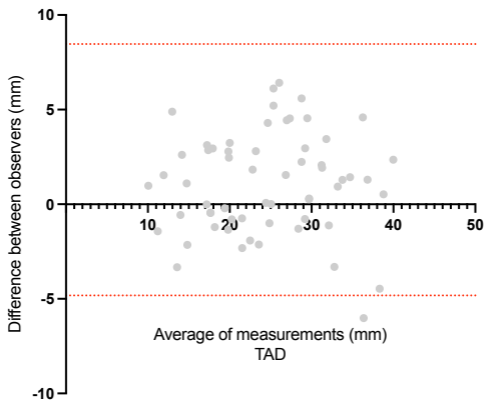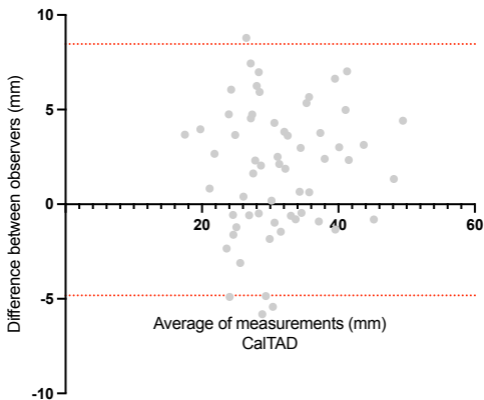

Supplement: Supplementary file 1 — Supplementary file1 (PDF 65 KB) [file 402_2026_6257_MOESM1_ESM.pdf]
